# Metabolism of Flavonoids in Novel Banana Germplasm during Fruit Development

**DOI:** 10.3389/fpls.2016.01291

**Published:** 2016-08-30

**Authors:** Chen Dong, Huigang Hu, Yulin Hu, Jianghui Xie

**Affiliations:** Key Laboratory of Tropical Fruit Biology, Ministry of Agriculture, South Subtropical Crop Research Institute, Chinese Academy of Tropical Agricultural ScienceZhanjiang, China

**Keywords:** banana pulp, soluble flavonoids, bound flavonoids, fruit development, antioxidant activity

## Abstract

Banana is a commercially important fruit, but its flavonoid composition and characteristics has not been well studied in detail. In the present study, the metabolism of flavonoids was investigated in banana pulp during the entire developmental period of fruit. ‘Xiangfen 1,’ a novel flavonoid-rich banana germplasm, was studied with ‘Brazil’ serving as a control. In both varieties, flavonoids were found to exist mainly in free soluble form and quercetin was the predominant flavonoid. The most abundant free soluble flavonoid was cyanidin-3-*O*-glucoside chloride, and quercetin was the major conjugated soluble and bound flavonoid. Higher content of soluble flavonoids was associated with stronger antioxidant activity compared with the bound flavonoids. Strong correlation was observed between antioxidant activity and cyanidin-3-*O*-glucoside chloride content, suggesting that cyanidin-3-*O*-glucoside chloride is one of the major antioxidants in banana. In addition, compared with ‘Brazil,’ ‘Xiangfen 1’ fruit exhibited higher antioxidant activity and had more total flavonoids. These results indicate that soluble flavonoids play a key role in the antioxidant activity of banana, and ‘Xiangfen 1’ banana can be a rich source of natural antioxidants in human diets.

## Introduction

The general role of phenolic compounds in plant physiology and allelopathy has been known for several years (Treutter, 2001). Phenolics, including flavonoids, phenolic acids, tannins, stilbenes, and lignins, have been reported as beneficial components of functional food by nutritionists (Ross et al., 2009; Bataglion et al., 2015; Kraujalytė et al., 2015; Liu et al., 2015). The flavonoids are the most abundant polyphenols in human diets (Dai and Mumper, 2010; Tsamo et al., 2015), and the most common group of polyphenols in plants as well (Xi et al., 2014). They can be generally divided into different sub-classes: flavonols, flavones, flavanonols, flavanones, flavanols, isoflavones, chalcones, and anthocyanidins (Xiao et al., 2015, 2016). Previous reports showed that different solvents used for extraction can influence the compositions of flavonoids in extracts because the solubility of each flavonoid could differ in a given solvent (Lou et al., 2014). Flavonoids have also been found in the insoluble fraction and associated with dietary fiber in tomato peel and roselle tea (Wang et al., 2016). Flavonoids exhibit anti-inflammatory, anti-neoplastic, and hepatoprotective activities, as well as strong antioxidant capacity (Lewis et al., 1999; Xi et al., 2014; Sharma et al., 2015; Xie et al., 2015). The mechanism of antioxidant activity of flavonoids involves excited oxygen species or the direct scavenging of oxygen free radicals, as well as the inhibition of oxidative enzymes that generate these reactive oxygen species (Kang et al., 2011). The health benefits of flavonoids are well recognized (Xiao et al., 2015, 2016; Yin et al., 2015), which arouses increasing interest in researchers for the development of agronomically important food crops with optimized flavonoid levels and composition (Rodrigues et al., 2011).

Banana is one of the most important fruit crops in the world (Yue-Lian and Qing-Fang, 2011), and serves as part of a well-balanced human diet and a staple food for more than 400 million people in many tropical and subtropical countries (Sun et al., 2013; Feng et al., 2016), and their utilization can be expected to increase in the future (Pereira and Maraschin, 2015; Tsamo et al., 2015). Banana fruits have pleasant flavor and offer excellent nutritional value (Santos et al., 2010). It has been reported as an important source of phenolic compounds, with the flavonoids being the major form (Schieber et al., 2001; Vijayakumar et al., 2008). However, few data have been reported regarding flavonoid metabolism in banana. To our knowledge, metabolism in banana pulp has not been reported and changes of different categories of flavonoids across development in banana have not been analyzed thus far. This study aimed to the change of flavonoid content in banana pulp by identifying and quantifying soluble and insoluble flavonoids in banana pulp at different stages of fruit development and to investigate the antioxidant activities of individual flavonoids and their potential health effects.

## Materials and Methods

### Sample Preparation

Banana fruits (*Musa* spp. AAB cv. ‘Xiangfen 1’ and *Musa* spp. AAA cv. ‘Brazil’) were obtained from a banana plantation in China (Zhanjiang, China) (**Supplementary Figures S1** and **S2**). Three uniform fruits (each fruit was a replicate) were randomly sampled at different development stages according to the cut-off bud days (days 5, 25, 45, 65, 85, and 85 + 3). These fruits were transferred to the laboratory within half an hour of collection. The tissues were immediately frozen in liquid nitrogen after sampling and stored at -80°C until further use. All analyses were performed in triplicate.

### Extraction of Soluble Phenolic Compounds

The levels of soluble phenolic compounds were determined using a modified method (Butsat et al., 2009). Soluble phenolic compounds refer to free and conjugated phenolic compounds in banana pulp. In brief, 2 g of each sample was extracted with 80% methanol (3 mL × 30 mL, 30 min each) through ultrasonication. Each extract was pooled and evaporated at 45°C to 10 mL under reduced pressure. The combined solution was extracted three times with the extraction solvent (ethyl acetate:diethyl ether = 1:1). The aqueous phase was collected for the conjugated phenolic compounds, whereas the organic phase was used to extract free phenolic compounds. For conjugated phenolic compounds, 0.9 mL of hydrochloric acid was added to the aqueous phase; the mixture was extracted three times with the extraction solvent (ethyl acetate:diethyl ether = 1:1) and was then lyophilized to dryness. The residue was dissolved in 2 mL of methanol and subjected to HPLC analysis. For free phenolic compounds, the organic phase was lyophilized to dryness, and the residue was dissolved in 2 mL of methanol and subjected to HPLC analysis.

### Extraction of Bound Phenolic Compounds

The bound phenolics were extracted according to the method described previously by Adom and Liu (2002) with minor modifications. In brief, the residue from the soluble fractions described above was drained and hydrolyzed directly with 2.5 M sodium hydroxide at room temperature for 12 h with shaking under nitrogen gas. The resulting solution was neutralized with an appropriate amount of hydrochloric acid and extracted with hexane to remove the lipids. The final solution was extracted three times with the extract (ethyl acetate:diethyl ether = 1:1). The organic phase was evaporated to dryness. Phenolic compounds were dissolved with 2 mL of methanol and analyzed using HPLC.

### HPLC Analysis of Flavonoids

Identification and quantification of banana flavonoids were done using the following method (Dykes et al., 2011). Flavonoids were separated using a SBC-18 (250 mm × 4 mm, 5 μm) column from Agilent (USA). The column temperature was maintained at 35°C. The injection volume was 20 μl. The mobile phase consisted of 4% formic acid in water (v/v) (Solvent A) and acetonitrile (Solvent B). The solvent flow rate was 1.0 ml/min. The 3-deoxyanthocyanidins were separated using the following gradients: 0–20 min, 12–20% B; 20–40 min, 20–50% B; 40–50 min, 50% B. Flavones and flavanones were separated using different gradient: 0–45 min, 15–41% B; 45–50 min, 41% B. The 3-deoxyanthocyanidins, flavones, and flavanones were detected at 485, 340, and 280 nm, respectively. Flavonoids were identified based on the retention times of commercial standards (**Supplementary Figure S3**), UV–Vis spectra, and data reported in the literature (Lewis et al., 1999). Quantification of each compound was accomplished by comparing the peak areas with that of a calibration curve of each standard.

### Determination of Total Flavonoids

Total flavonoid level was tested with Folin–Ciocalteu’s phenol reagent (Bouayed et al., 2011). One gram of each sample was extracted with 3 mL 95% methanol, and then the solution was added to a 25 mL volumetric flask containing 9 mL of water. Then, 1 mL of Folin–Ciocalteu’sphenol reagent was added to the mixture and shaken. After 5 min, 10 mL of 7% aqueous Na_2_CO_3_ solution was added. The solution was then immediately diluted to a final volume of 25 mL with water and mixed thoroughly. After incubation for 30 min at 40°C, the absorbance versus the prepared blanks was read at 760 nm. Total phenolic content in banana was defined as milligrams of gallic acid equivalents (GAE) 1 g of fresh weight of sample.

### Antioxidant Capacity

Antioxidant capacity was detected according to a previous method (Boulekbache-Makhlouf et al., 2013), with modifications. About 1 mL of the sample extract was mixed with 1 mL of phosphate buffer (0.2 M, pH 6.6) and 1 mL of 1% potassium ferricyanide [K_3_Fe(CN)_6_]. The mixture was incubated at 50°C for 20 min. Trichloroacetic acid (1 mL, 10%) was added to the solution, which was then centrifuged at 3000 × *g* for 10 min. The supernatant was gathered and mixed with distilled water (1.5 mL) and FeCl_3_ (150 μL, 0.1%), and the absorbance was measured at 700 nm. The mean of absorbance values was plotted against concentration, and a linear regression analysis was carried out. Ascorbic acid was used as the positive control.

### Soluble Sugar Content and Fruit Weight

Soluble sugar content in the pulp extracts was determined using the anthrone method of Yemm and Willis (1954). Fruit weight was recorded using an electronic scale (METTLER TOLEDO, ME303).

### Chemicals

All flavonoids standards (purity ≥ 98%, HPLC) were purchased from Sigma–Aldrich (St. Louis, MO, USA).

### Statistical Analysis

Data were analyzed statistically by ANOVA, and differences in means were evaluated using Duncan’s new multiple range test (*P* < 0.05) with SPSS version 16.0 (SPSS Inc., Chicago, IL, USA). Pearson’s correlations were calculated to determine relationship among the measured variables.

## Results

### Quercetin

**FIGURE 1:**
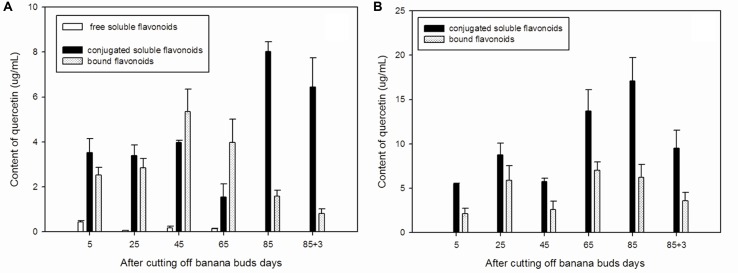
**Changes of quercetin levels in banana pulp during the development stage.** **(A)** ‘Brazil’ banana, **(B)** ‘Xiangfen 1’ banana.

The free soluble quercetin is relatively, whereas the conjugated soluble quercetin is abundant low in ‘Brazil’ banana (**Figure 1A**), with the highest level of free soluble quercetin observed on day 5. The lowest conjugated soluble quercetin level was observed on day 65, and the highest level was noted on day 85. Levels of bound quercetin increased during the early developmental period, reached its maximum level on day 45, and then decreased gradually afterward.

In ‘Xiangfen 1’ banana (**Figure 1B**), free soluble quercetin was undetected during the entire developmental period. It predominantly expressed the conjugated soluble quercetin.

In comparison, conjugated soluble quercetin and bound quercetin were the two main forms of quercetin and levels of conjugated soluble quercetin peaked on day 85 in both ‘Brazil’ and ‘Xiangfen 1’ banana. ‘Xiangfen 1’ exhibited twofold higher conjugated soluble quercetin content at the late developmental period compared with ‘Brazil.’ The total quercetin levels in ‘Xiangfen 1’ were higher than those in ‘Brazil’ at all stages, except for day 45. Collectively, these data suggested that conjugated soluble quercetin might be the main flavonoid in banana.

### Cyanidin-3-*O*-Glucoside Chloride

**FIGURE 2:**
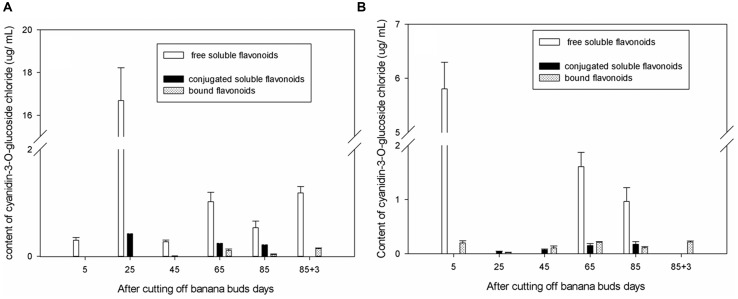
**Changes of cyanidin-3-*O*-glucoside chloride levels in banana pulp during the development stage.** **(A)** ‘Brazil’ banana, **(B)** ‘Xiangfen 1’ banana.

As shown in **Figure 2A**, in ‘Brazil’ banana, the level of free soluble cyanidin-3-*O*-glucoside chloride were significantly higher than those of other forms of cyanidin-3-*O*-glucoside chloride (*P* < 0.05), and the level of bound cyanidin-3-*O*-glucoside chloride were the lowest. The level of free soluble cyanidin-3-*O*-glucoside chloride peaked on day 25, which was more than 10-fold higher than those on other days (*P* < 0.05). The highest level of conjugated soluble cyanidin-3-*O*-glucoside chloride was also observed on day 25. The bound cyanidin-3-*O*-glucoside chloride was not obtained till day 65, with its highest level shown on day 88 and the lowest level on day 85.

In ‘Xiangfen 1’ banana (**Figure 2B**), no free soluble cyanidin-3-*O*-glucoside chloride was detected on days 25, 45, and 88. The greatest level of free soluble cyanidin-3-*O*-glucoside chloride was found at the initial stage, and the lowest level was observed on day 85. The levels of both the conjugated soluble and bound cyanidin-3-*O*-glucoside chlorides were low during fruit development, with the conjugated soluble cyanidin-3-*O*-glucoside chloride increased gradually from day 25 to day 85 and the bound cyanidin-3-*O*-glucoside chloride peaked on day 88.

The level of cyanidin-3-*O*-glucoside chloride in ‘Brazil’ was significantly higher (*P* < 0.05) than those in ‘Xiangfen 1’ on days 25 and 45, but the level in ‘Xiangfen 1’ sharply increased by day 65 and were greater than those in ‘Brazil’ banana on days 65 and 85. Overall, the cyanidin-3-*O*-glucoside chloride mainly exists in free soluble form in banana fruits of both varieties, but its level is relatively higher in ‘Brazil’ than ‘Xiangfen 1.’

### Ellagic Acid

**FIGURE 3:**
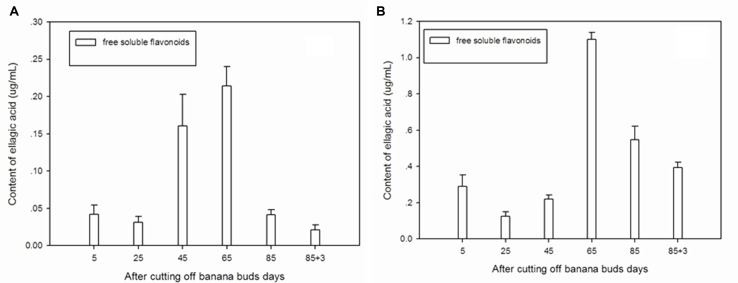
**Changes of ellagic acid levels in banana pulp during the development stage.** **(A)** ‘Brazil’ banana, **(B)** ‘Xiangfen 1’ banana.

**Figure 3A** showed that free soluble ellagic acid was the main form of ellagic acid in ‘Brazil’ banana. The level of free soluble ellagic acid was very low during the initial period, but greatly increased after day 25, peaking on day 65.

For the ‘Xiangfen 1’ banana (**Figure 3B**), the free soluble ellagic acid was the sole form of ellagic acid. The free soluble ellagic acid content was lower at the early stage compared with those at the later stage. The greatest level of free soluble ellagic acid was found on day 65, after that it gradually decreased. The level of ellagic acid in ‘Xiangfen 1’ banana were higher than that in ‘Brazil’ banana at all stages.

### Catechin

**FIGURE 4:**
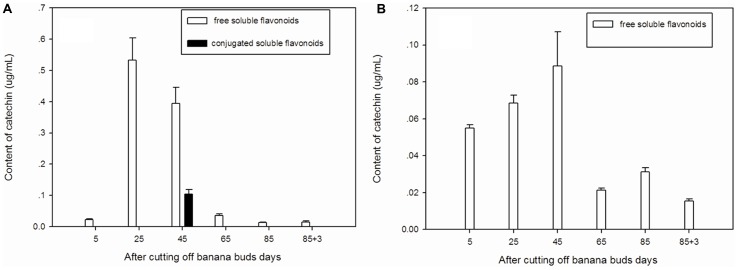
**Changes of catechin levels in banana pulp during the development stage.** **(A)** ‘Brazil’ banana, **(B)** ‘Xiangfen 1’ banana.

Free soluble catechin was the major type of catechin in ‘Brazil’ (**Figure 4A**). The highest level of free soluble catechin was observed on day 25 and then gradually decreased from day 25 to day 85. Conjugated soluble catechin was detected only onday 45.

The catechin in ‘Xiangfen 1’ was present mainly in free soluble form (**Figure 4B**), which gradually increased from day 5 to day 45 but after that it decreased quickly till day 65. Thus, the free soluble catechin level was significantly higher (*P* < 0.05) on day 45 than day 65. In fruits of both cultivars, high level of catechin was found on days 25 and 45, with the ‘Brazil’ showing a higher catechin level.

### Gallocatechin

**FIGURE 5:**
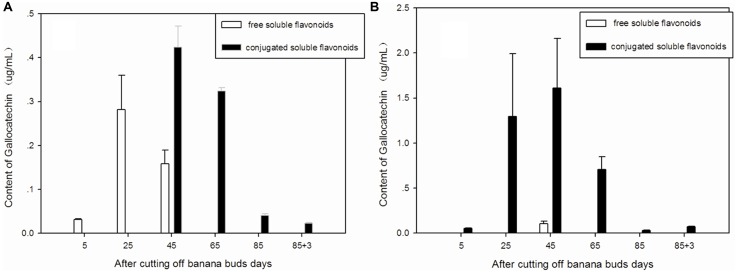
**Changes of gallocatechin levels in banana pulp during the development stage.** **(A)** ‘Brazil’ banana, **(B)** ‘Xiangfen 1’ banana.

Free and conjugated soluble gallocatechin were the main types of gallocatechin in ‘Brazil’ banana (**Figure 5A**), as no bound gallocatechin was observed at any stage. The free soluble gallocatechin in ‘Brazil’ banana was observed on days 5, 25, and 45, and its concentration was ninefold higher on day 25 than that on day 5. The conjugated soluble gallocatechin was detected from day 45 to day 88, with the highest level found on day 65.

The conjugated soluble form of gallocatechin was dominant in ‘Xiangfen 1’ (**Figure 5B**), with its highest level observed on day 45. Its level was significantly higher than the free soluble gallocatechin level on the same day (*P* < 0.05).

The highest level of gallocatechin in both types of banana was found on day 45. ‘Xiangfen 1’ showed a higher level of gallocatechin ‘Brazil’ at all stages except day 85. These observations reveal that the middle developmental period is an important stage for gallocatechin formation in banana.

### Soluble and Bound Flavonoids

**FIGURE 6:**
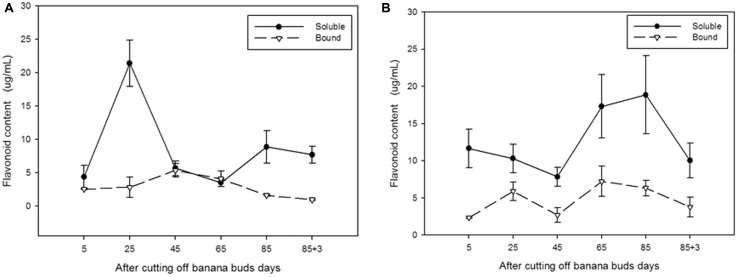
**The contents of soluble and bound flavonoids in ‘Brazil’ banana **(A)** and ‘Xiangfen 1’ banana **(B)****.

As shown in **Figure 6A**, the soluble flavonoid content in ‘Brazil’ peaked on day 25 (21.40 μg/mL) and then gradually decreased till day 65 to reach the lowest level (3.50 μg/mL). For bound flavonoids, the highest and lowest contents were observed on days 45 and 88, respectively. The soluble content was evidently higher than the bound content on days 25, 85, and 88 (*P* < 0.05).

In contrast, the content of soluble flavonoid in ‘Xiangfen 1’ (**Figure 6B**) peaked on day 85 (18.85 μg/mL), and the highest content of bound flavonoid was observed on day 65 (7.23 μg/mL). The content of soluble flavonoids were evidently greater than that of bound flavonoids in the entire developmental period (*P* < 0.05). These results show that most of the flavonoids in banana pulp are soluble.

### Soluble Sugar

**FIGURE 7:**
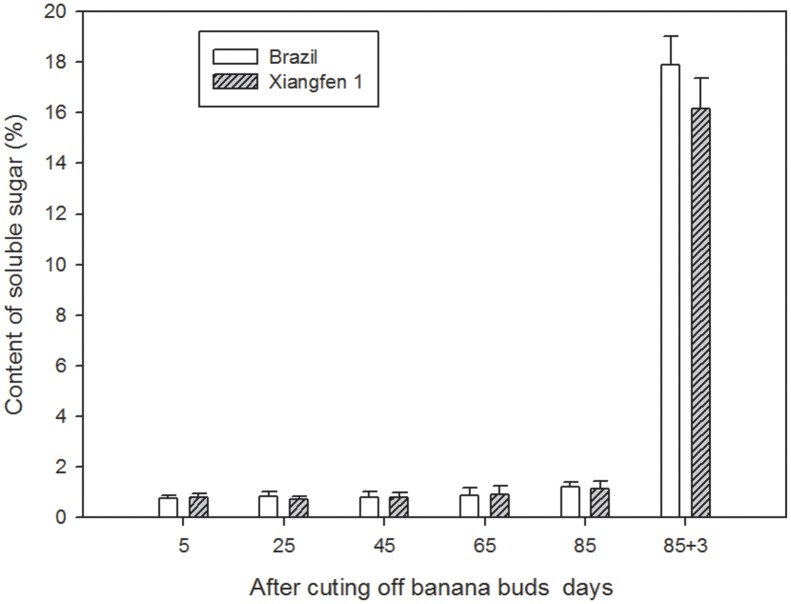
**Changes of soluble sugar levels in banana pulp during the development stage**.

The soluble sugar content was low in both types of fruits (before day 85, **Figure 7**), and there were no significant differences between them. However, the content increased sharply (from 1.2 to 17.9%) during the harvesting period (after day 85), with ‘Brazil’ showing a higher level than ‘Xiangfen 1’ on day 88, indicating the harvesting period is a crucial time for accumulation of soluble sugar content in banana.

### Fruit Weight

**FIGURE 8:**
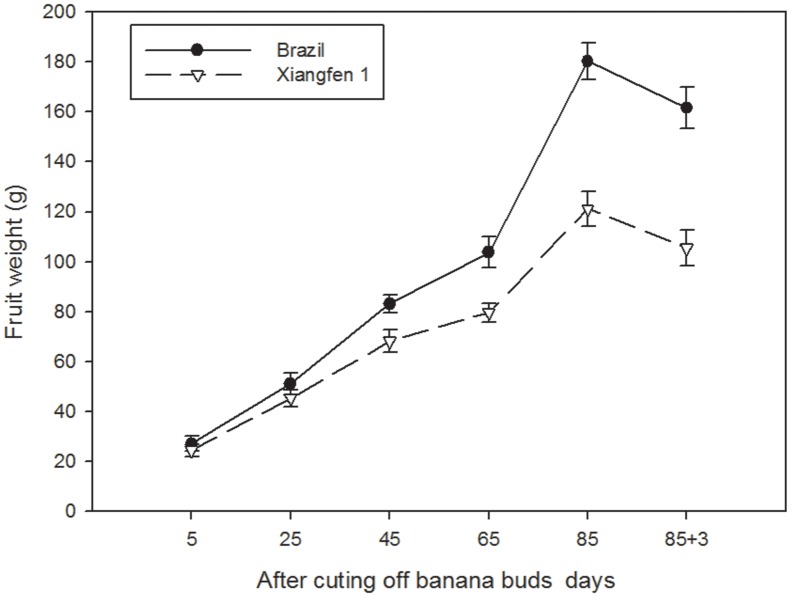
**Changes of fruit weight in banana pulp during the development stage**.

As shown in **Figure 8**, the fruit weight in the two types of banana increased gradually with fruit development, and decreased after day 85. The fruit weight in ‘Brazil’ was significantly higher than that in ‘Xiangfen 1’ from day 45 to day 88 (*P* < 0.05). However, from day 5 to day 25, no significant difference was detected between the two types of banana. These findings reveal that difference in fruit weight among different types of banana is likely determined in the later developmental period.

### Antioxidant Capacity of Soluble and Bound Flavonoids

**FIGURE 9:**
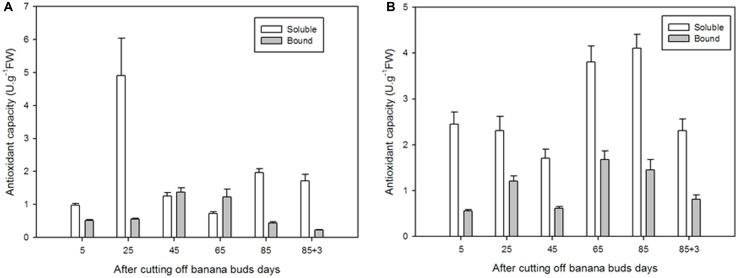
**The antioxidant capacities of soluble and bound flavonoids in ‘Brazil’ banana **(A)** and ‘Xiangfen 1’ banana **(B)****.

For soluble flavonoids in ‘Brazil’, as shown in **Figure 9A**, the extracts on day 25 exhibited significantly higher antioxidant capacity than those on other days (*P* < 0.05), with the lowest antioxidant capacity showed on day 65 (0.72 U⋅g^-1^ FW). For bound flavonoids, the antioxidant capacity increased from day 5 to day 45 and then decreased till day 88. In comparison, the soluble flavonoids showed greater antioxidant capacity the bound ones at all stages except for days 45 and 65.

The antioxidant capacity of ‘Xiangfen 1’ fruit was evaluated as well (**Figure 9B**). For soluble flavonoids, the antioxidant capacity peaked and bottomed on day 85 (4.11 U⋅g^-1^ FW) and on day 45 (1.71 U⋅g^-1^ FW), respectively. For bound flavonoids, the strongest (1.68 U⋅g^-1^ FW) and lowest antioxidant capacities (0.56 U⋅g^-1^ FW) were observed on days 85 and 5, respectively. Similarly, the soluble flavonoids had higher antioxidant capacities compared with the bound extracts at all stages. These results indicate that soluble flavonoids are probably the major antioxidants in banana.

### Total Flavonoid and Total Antioxidant Capacity (TAC)

**FIGURE 10:**
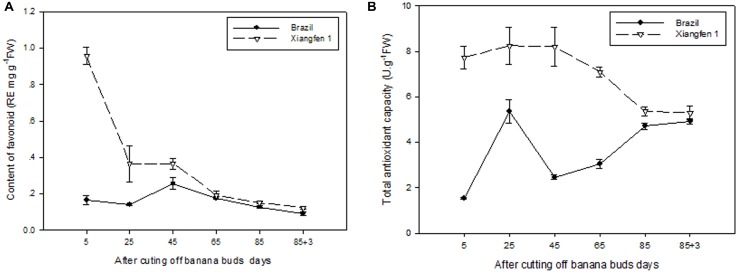
**Changes of total flavonoid level **(A)** and total antioxidant capacity **(B)** in banana pulp during the development stage**.

As shown in **Figure 10A**, the level of total flavonoid gradually decreased with fruit development in ‘Xiangfen 1.’ The highest total flavonoid level was detected on day 5 and then decreased quickly till day 25. Consequently, a significantly lower total flavonoid level (*P* < 0.05) was observed on day 25 compared with that on day 5 in ‘Xiangfen 1.’ For the ‘Brazil’ fruits, the level of total flavonoid increased at the beginning and peaked on day 45, and gradually decreased thereafter. The total flavonoid level was significantly higher in ‘Xiangfen 1’ than that in ‘Brazil’ during the early developmental stage.

The TAC in ‘Xiangfen 1’ slowly increased at the beginning and then gradually decreased from day 25 to day 88 (**Figure 10B**). As a result, the highest TAC was detected on day 25, and the lowest TAC on day 88. The TAC fluctuated in the ‘Brazil’ fruits, with the highest TAC observed on day 25 and the lowest TAC on day 5.

The TAC in ‘Xiangfen 1’ was significantly higher than that in ‘Brazil’ in the first 65 days. No significant difference of TAC was found between them during the later period of development. These findings reveal that increase in flavonoids, both in soluble and bound forms, might contribute to increase in antioxidant activity in banana.

### Correlation

Pearson correlations were calculated for the objective variables measured during banana development (**Tables 1** and **2**). The results showed statistically significant correlations (*r* = 0.813) between TAC and the level of cyanidin-3-*O*-glucoside chloride (*P* < 0.05; **Table 1**), which indicated that cyanidin-3-*O*-glucoside chloride likely plays a major role in the antioxidative activity in ‘Brazil’ banana. For ‘Xiangfen 1’ (**Table 2**), TAC was highly correlated with the flavonoid content (*r* = 0.862), which suggested that flavonoid was the main antioxidative component in the pulp. Significant correlations were also found between the flavonoid content and cyanidin-3-*O*-glucoside chloride level (*r* = 0.834). Taken together, these results illustrate that cyanidin-3-*O*-glucoside chloride, as a main type of flavonoids, is one of the major antioxidants in banana.

**Table 1:** Pearson correlations among total antioxidant capacity (TAC) and flavonoid of pulp during the development of ‘Brazil’ banana.

	TAC	Flavonoid	Quercetin	Cyanidin-3-*O*-glucoside chloride	Ellagic acid	Catechin	Gallocatechin
TAC	1	-0.665	0.058	0.813^∗^	-0.481	0.386	-0.748
Flavonoid	-0.665	1	0.267	-0.189	0.695	0.167	0.987
Quercetin	0.058	0.267	1	-0.469	-0.085	0.580	0.958
Cyanidin-3-*O*-glucoside chloride	0.813^∗^	-0.189	-0.469	1	-0.401	0.504	-0.655
Ellagic acid	-0.481	0.695	-0.085	-0.401	1	-0.768	0.358
Catechin	0.386	0.167	0.580	0.504	-0.768	1	0.323
Gallocatechin	-0.748	0.987	0.958	-0.655	0.358	0.323	1


*^∗^Significant difference at *P* < 0.05.*

**Table 2:** Pearson correlations among TAC and flavonoid of pulp during the development of ‘Xiangfen 1’ banana.

	TAC	Flavonoid	Quercetin	Cyanidin-3-*O*-glucoside chloride	Ellagic acid	Catechin	Ggallocatechin
TAC	1	0.862^∗^	-0.551	0.568	-0.339	0.754	0.897
Flavonoid	0.862^∗^	1	-0.676	0.834^∗^	-0.391	-0.802	0.914
Quercetin	-0.551	-0.676	1	-0.286	0.662	-0.019	-0.994
Cyanidin-3-*O*-glucoside chloride	0.568	0.834^∗^	-0.286	1	0.101	-0.793	-0.888
Ellagic acid	-0.339	-0.391	0.662	0.101	1	-0.315	-0.871
Catechin	0.754	-0.802	-0.019	-0.793	-0.315	1	0.654
Gallocatechin	0.897	0.914	-0.994	-0.888	-0.871	0.654	1


*^∗^Significant difference at *P* < 0.05.*

## Discussion

Flavonoids are the largest group of polyphenolic plant secondary metabolites (Rodrigues et al., 2011). Different subclasses of flavonoids exist in banana fruits (Tsamo et al., 2015), and flavonoids of different banana varieties share some characteristics (Bennett et al., 2010). In this study, five flavonoids (quercetin, cyanidin-3-*O*-glucoside chloride, ellagic acid, catechin, and gallocatechin) were detected in both banana varieties at all fruit developmental stages. This result is in agreement with a previous study (Someya et al., 2002), which also identified gallocatechin and anthocyanin in banana fruits. However, rutin and epicatechin were only found at some stages in both banana varieties, which is consistent with a previous study by Bennett et al. (2010) on banana fruit. It has been recognized that some fruits contain abundant quercetin (Kuti, 2004), which is one of the most commonly consumed flavonoids that has been studied for its potential health benefits (Kuti, 2004; Tsamo et al., 2015). In this study, quercetin was the predominant flavonoid at all stages in both types of banana. Moreover, the quercetin content was the highest among conjugated soluble flavonoids and bound flavonoids. Free soluble flavonoids were the main types of flavonoids in ‘Xiangfen 1’ banana and ‘Brazil’ banana. This finding agreed with the results of Lou et al. (2014), who reported that the free soluble flavonoid content was significantly higher than that of other flavonoids in calamondin. Most of phenolic compounds with antioxidant activity in jujube existed as the free form (Wang et al., 2016).

Diverse flavonoid traits were found in different banana varieties (Bennett et al., 2010; Tsamo et al., 2015). In this study, the total quercetin, total ellagic acid, and gallocatechin contents of ‘Xiangfen 1’ banana during the entire developmental period were higher than those in ‘Brazil’ banana, whereas the cyanidin-3-*O*-glucoside chloride and catechin contents were lower in ‘Xiangfen 1’ banana than in ‘Brazil’ banana. A similar study report significant differences in total flavonols concentration of onion were observed between varieties (Rodrigues et al., 2011). Flavonoids have been shown to exhibit strong antioxidant capacities (Kang et al., 2011; Lou et al., 2015; Sait et al., 2015; Yang et al., 2015). In the present study, the total flavonoid content of ‘Xiangfen 1’ banana was higher than that of ‘Brazil’ banana during the first 45 days. During the same period, the antioxidant capacity of ‘Xiangfen 1’ banana was also stronger than that of ‘Brazil’ banana. This result is in agreement with the study of Vijayakumar et al. (2008), who found that flavonoids from banana act as effective antioxidants. Another similar study showed that NO treatment significantly enhanced flavonoid contents in the mushrooms and the antioxidant activities in the NO-fumigated mushrooms were highly correlated with the contents of flavonoids (Dong et al., 2012). Study has shown that anthocyanins are the largest and most important group of water-soluble and vacuolar pigments in nature (Cavalcanti et al., 2011) and it have been identified in wild banana bracts (Kitdamrongsont et al., 2008). In the present study, the major free soluble flavonoid was cyanidin-3-*O*-glucoside chloride in banana pulp. Anthocyanins are pigments with a wide range of biological effects, including antioxidant activity (Cavalcanti et al., 2011; Li et al., 2014; Lee et al., 2015). The present results showed that cyanidin-3-*O*-glucoside chloride level was closely related to the antioxidant capacity of ‘Brazil’ banana, and the total flavonoid content was significantly correlated with the antioxidant capacity of ‘Xiangfen 1’ banana. Moreover, significant correlation was found between cyanidin-3-*O*-glucoside chloride content and total flavonoid content in ‘Xiangfen 1’ banana, suggesting that cyanidin-3-*O*-glucoside chloride is responsible for the antioxidant activity of banana. A similar result was also reported for ‘dabai’ fruits, which contain phenolic compounds (including flavonoids and anthocyanin) and exhibit strong antioxidant activity (Chew et al., 2011). However, further studies need to be performed to evaluate the role of these individual flavonoids that contribute to antioxidant activity.

## Conclusion

In this study, the flavonoid profiles of banana pulp during the developmental period were monitored from the fruitlet stage to the fully ripe stage. Quercetin was the predominant flavonoid in the tested banana varieties and also the main conjugated soluble and bound flavonoid. The majority of the flavonoids were present in free soluble form, and cyanidin-3-*O*-glucoside chloride was the dominant free soluble flavonoid in all pulp extracts. High antioxidant activity was correlated to the levels of total flavonoid and cyanidin-3-*O*-glucoside chloride. The total flavonoid content was higher in ‘Xiangfen 1’ banana than that in the control variety, which is consistent with the antioxidant activity profile of ‘Xiangfen 1’ banana. Fruit weight and soluble sugar content in both varieties increased quickly during the later developmental period. Our results revealed that the levels and compositions of flavonoids vary considerably during the growth and development of banana. The observed levels of flavonoid, especially cyanidin-3-*O*-glucoside chloride, and the antioxidant properties of pulp extracts indicate that banana pulp can be a valuable source of antioxidant-rich nutraceuticals.

## Author Contributions

JX, HH, and CD conceived and designed the experiments. CD and YH performed the experiments and helped with the data analysis. HH and CD wrote the paper. All authors read and approved the final manuscript.

## Conflict of Interest Statement

The authors declare that the research was conducted in the absence of any commercial or financial relationships that could be construed as a potential conflict of interest.

## Supplementary Material

**FIGURE S1 | ‘Brazil’ banana and the orange pulp banana variety ‘Xiangfen 1’.**

**FIGURE S2 | The banana in different developmental stages.**

**FIGURE S3 | HPLC chromatogram of standard flavonoids (A) and sample extract (B).**
