# Supplementary figures and images for: Metabolism of Flavonoids in Novel Banana Germplasm during Fruit Development
Source: Front Plant Sci. 2016 Aug 30;7:1291. doi: 10.3389/fpls.2016.01291 (PMC5003829; doi:10.3389/fpls.2016.01291)

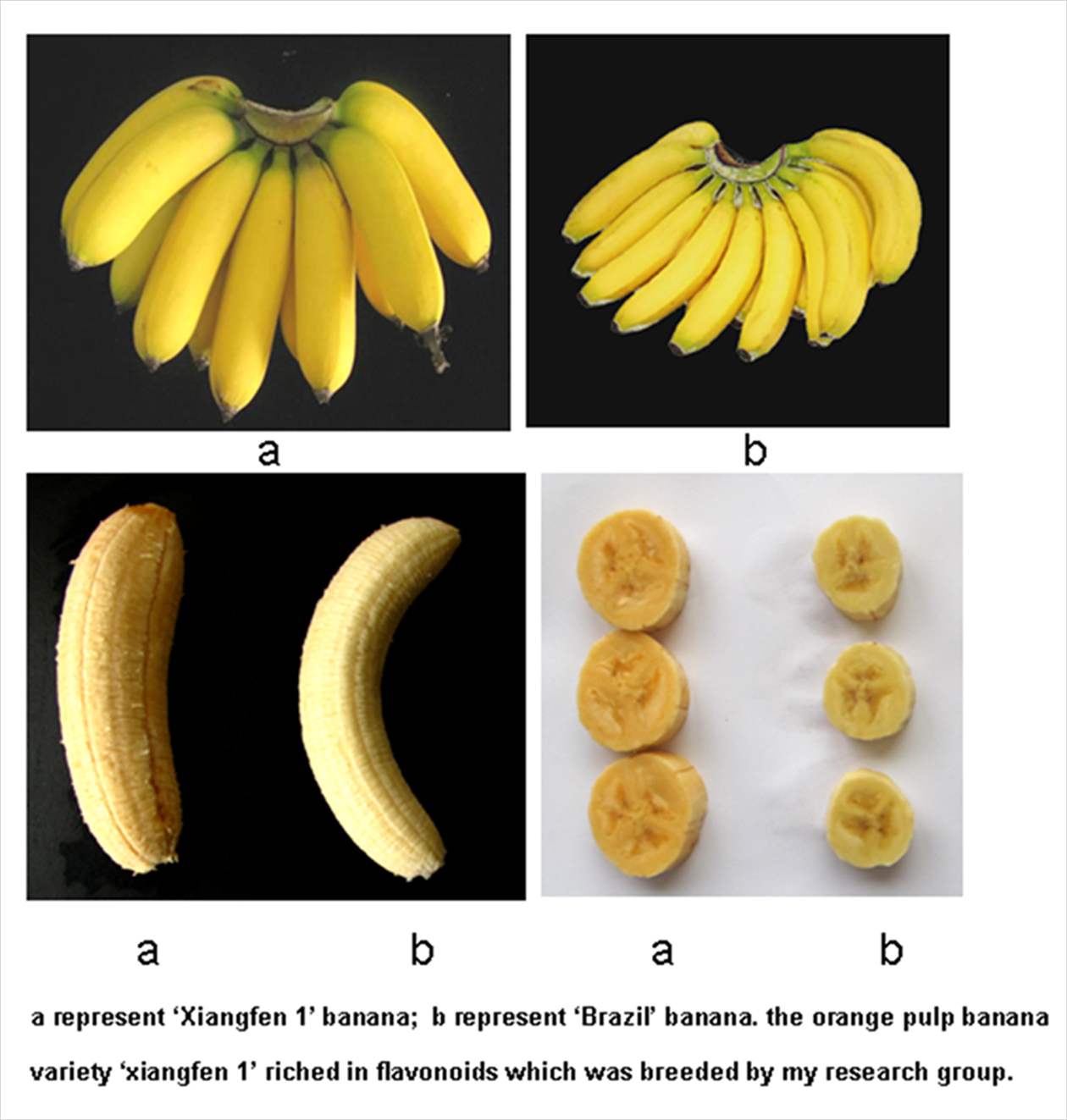

Supplement: Supplementary file 1 [file Image_1.TIF]

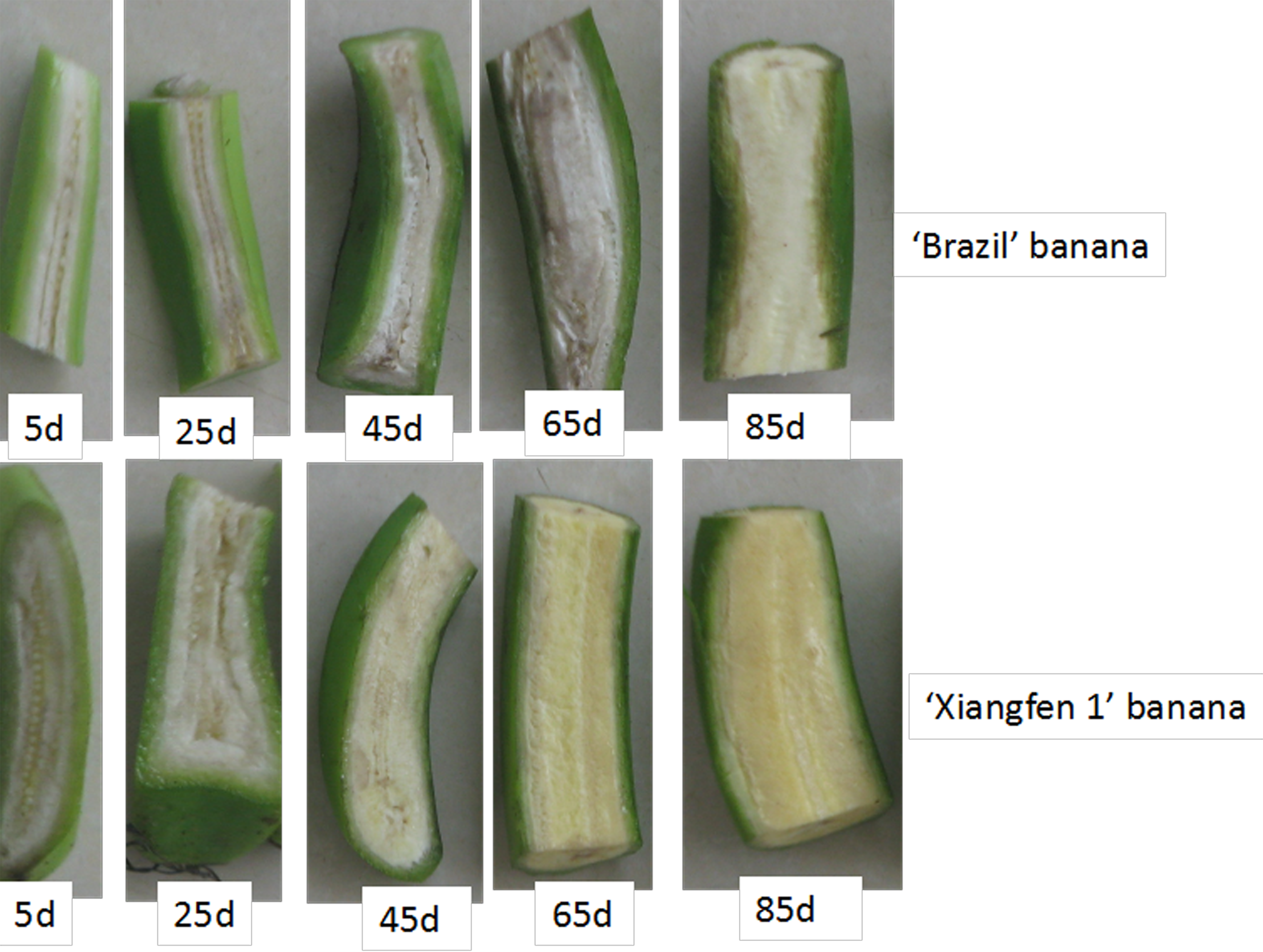

Supplement: Supplementary file 2 [file Image_2.TIF]

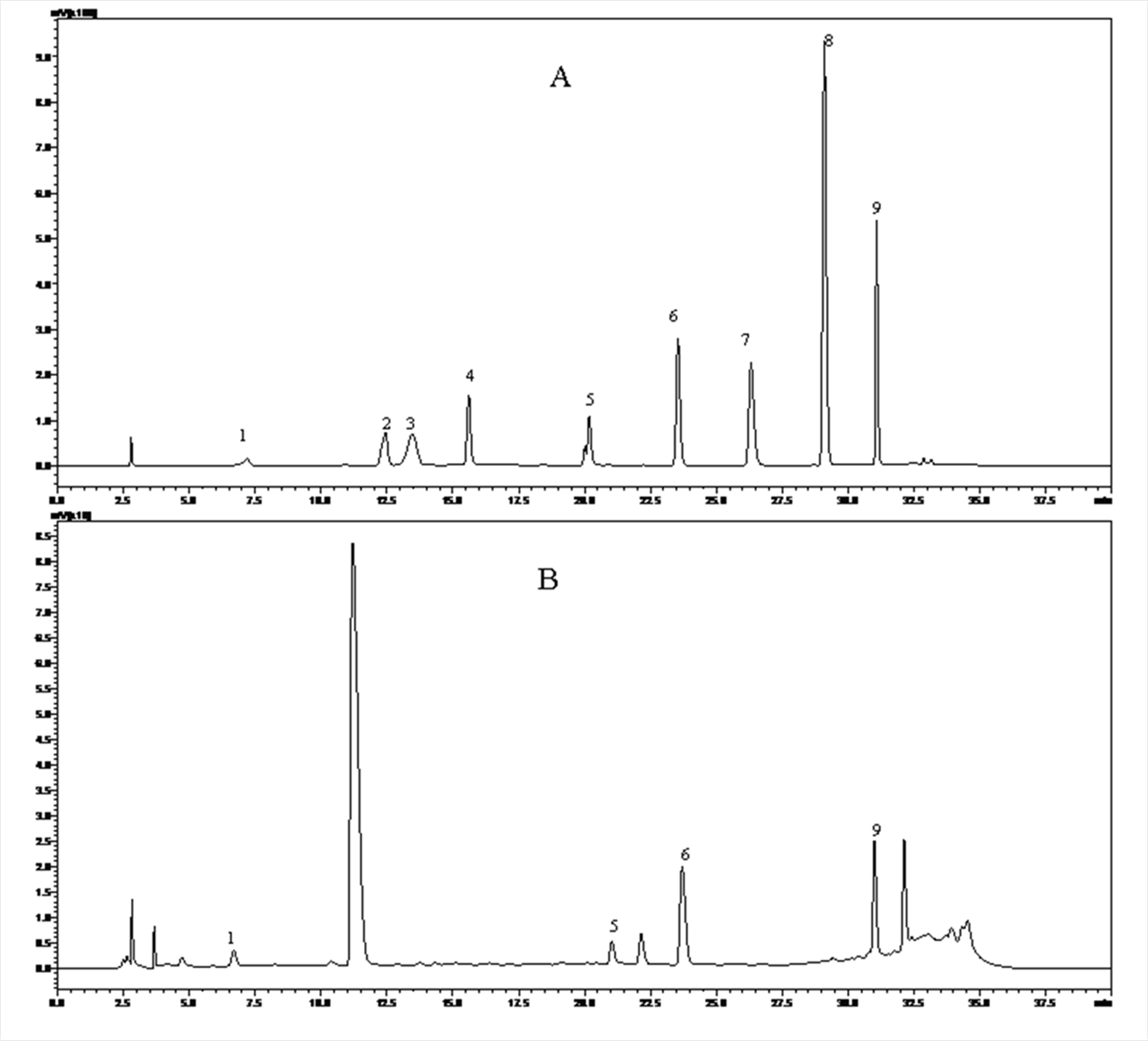

Supplement: Supplementary file 3 [file Image_3.TIF]
